# Supplementary material for: Re-evaluating heart rate variability biomarkers for glucose sensing: the impact of age normalisation and subject-independent validation
Source: BMC Med Inform Decis Mak. 2026 Apr 1;26:166. doi: 10.1186/s12911-026-03455-8 (PMC13169770; doi:10.1186/s12911-026-03455-8)
Supplement: Supplementary file 1 — Supplementary Material 1 [file 12911_2026_3455_MOESM1_ESM.docx]

**Supplementary Materials:** Re-evaluating Heart Rate Variability Biomarkers for Glucose Sensing: The Impact of Age Normalisation and Subject-Independent Validation

**Authors:** Md Basit Azam^1^*, Sarangthem Ibotombi Singh^1^

**Affiliations:** ^1^Department of Computer Science & Engineering, School of Engineering, Tezpur University, Napaam – 784 028, Tezpur, Assam, India.

**Corresponding Author:** Md Basit Azam ([mdbasit@tezu.ernet.in](mailto:mdbasit@tezu.ernet.in))

**1. Cohort Selection**


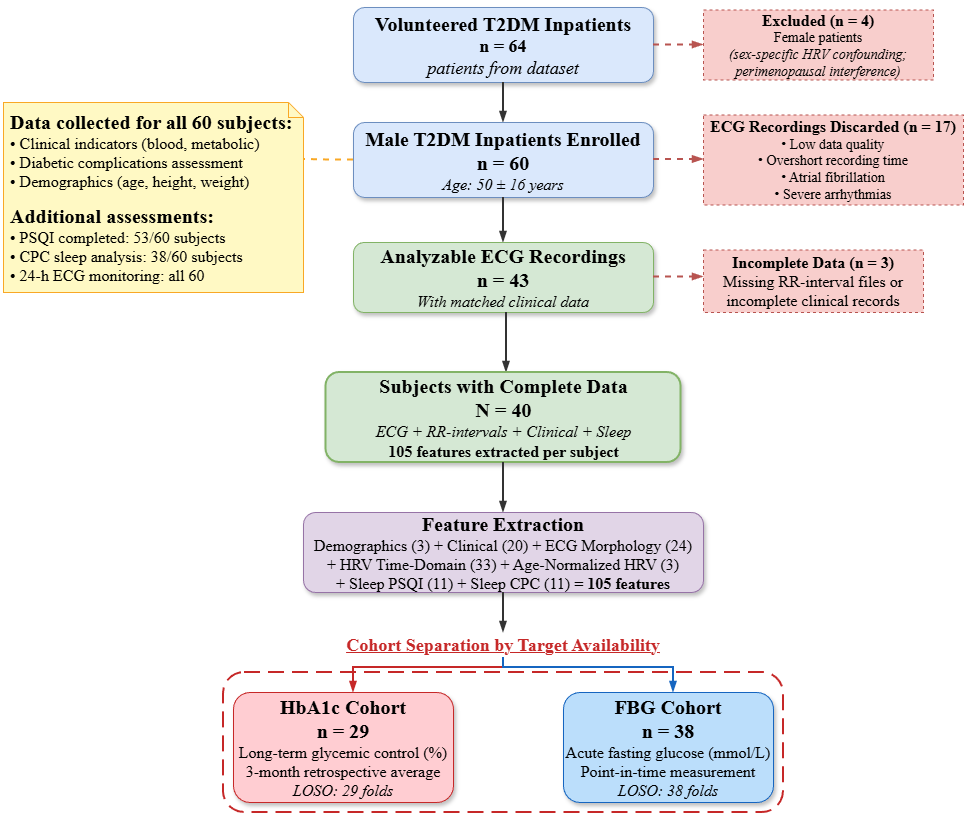


**Figure 1** Participant flow diagram

Figure 1 shows that of 64 volunteered T2DM inpatients, 4 were excluded (female), and 17 ECG recordings were discarded (low quality, arrhythmias), yielding 43 analysable subjects. After removing 3 subjects with incomplete data, 40 subjects with complete feature sets were assigned to HbA1c (n = 29) and FBG (n = 38) cohorts based on target availability.

# Table S1: Feature Domain Summary

A total of 105 features were extracted across 7 domains from the Cheng et al. (2023) dataset. Features #84–86 (psqi_age, psqi_height, psqi_weight) are duplicate demographics loaded from the PSQI form and retained as-is from the original data source. Target variables (HbA1c, FBG), gender, and diabetic complication indicators were excluded from the modelling feature set. Three PSQI-form demographics (psqi_age, psqi_height, psqi_weight) duplicate the Demographics domain but were retained as extracted.

| **Feature Domain** | **Count** | **Source File(s)** |
| --- | --- | --- |
| Demographics | 3 | Clinical indicators.xlsx |
| Clinical Measurements | 20 | Clinical indicators.xlsx |
| ECG Morphology | 24 | ECG .mat files (all/sleep/day) |
| HRV Time-Domain | 33 | RR-interval .mat files (DS/RS/REM) |
| Age-Normalized HRV | 3 | Derived from HRV + age |
| Sleep Quality (PSQI) | 11 | Objective sleep quality.xlsx |
| Sleep Quality (CPC) | 11 | Subjective sleep quality.xlsx |
| **Total** | **105** |  |

# Table S1 (continued): Complete Feature Inventory (105 Features)

| **Sl No.** | **Feature Name** | **Description** | **Domain** | **Source** |
| --- | --- | --- | --- | --- |
| 1 | age | Patient age (years) | Demographics | Clinical indicators |
| 2 | height | Patient height (cm) | Demographics | Clinical indicators |
| 3 | weight | Patient weight (kg) | Demographics | Clinical indicators |
| 4 | SBP (mmHg) | Systolic blood pressure | Clinical Measurements | Clinical indicators |
| 5 | DBP (mmHg) | Diastolic blood pressure | Clinical Measurements | Clinical indicators |
| 6 | WBC (×10⁹/L) | White blood cell count | Clinical Measurements | Clinical indicators |
| 7 | N% (%) | Neutrophil percentage | Clinical Measurements | Clinical indicators |
| 8 | Hb (g/L) | Hemoglobin | Clinical Measurements | Clinical indicators |
| 9 | PLT (×10⁹/L) | Platelet count | Clinical Measurements | Clinical indicators |
| 10 | CRP (mg/L) | C-reactive protein | Clinical Measurements | Clinical indicators |
| 11 | ALT (U/L) | Alanine aminotransferase | Clinical Measurements | Clinical indicators |
| 12 | AST (U/L) | Aspartate aminotransferase | Clinical Measurements | Clinical indicators |
| 13 | AST/ALT | De Ritis ratio | Clinical Measurements | Clinical indicators |
| 14 | GGT (U/L) | Gamma-glutamyltransferase | Clinical Measurements | Clinical indicators |
| 15 | BUN (mmol/L) | Blood urea nitrogen | Clinical Measurements | Clinical indicators |
| 16 | UA (mmol/L) | Uric acid | Clinical Measurements | Clinical indicators |
| 17 | TG (mmol/L) | Triglycerides | Clinical Measurements | Clinical indicators |
| 18 | HDL-C (mmol/L) | High-density lipoprotein cholesterol | Clinical Measurements | Clinical indicators |
| 19 | LDL-C (mmol/L) | Low-density lipoprotein cholesterol | Clinical Measurements | Clinical indicators |
| 20 | UMA (mg) | Urine micro-albumin | Clinical Measurements | Clinical indicators |
| 21 | UCr (g) | Urine creatinine | Clinical Measurements | Clinical indicators |
| 22 | UACR (mg/g) | Urine albumin-creatinine ratio | Clinical Measurements | Clinical indicators |
| 23 | Lower extremity atherosclerosis or stenosis | Complication indicator (0/1) | Clinical Measurements | Clinical indicators |
| 24 | ecg_all_length | Number of ECG samples (all recording) | ECG Morphology | ECG .mat files (all) |
| 25 | ecg_all_duration_hours | Recording duration in hours (all) | ECG Morphology | ECG .mat files (all) |
| 26 | ecg_all_mean | Mean ECG amplitude (all) | ECG Morphology | ECG .mat files (all) |
| 27 | ecg_all_std | Standard deviation of ECG amplitude (all) | ECG Morphology | ECG .mat files (all) |
| 28 | ecg_all_min | Minimum ECG amplitude (all) | ECG Morphology | ECG .mat files (all) |
| 29 | ecg_all_max | Maximum ECG amplitude (all) | ECG Morphology | ECG .mat files (all) |
| 30 | ecg_all_range | ECG amplitude range (all) | ECG Morphology | ECG .mat files (all) |
| 31 | ecg_all_snr_estimate | Signal-to-noise ratio estimate (all) | ECG Morphology | ECG .mat files (all) |
| 32 | ecg_sleep_length | Number of ECG samples (sleep recording) | ECG Morphology | ECG .mat files (sleep) |
| 33 | ecg_sleep_duration_hours | Recording duration in hours (sleep) | ECG Morphology | ECG .mat files (sleep) |
| 34 | ecg_sleep_mean | Mean ECG amplitude (sleep) | ECG Morphology | ECG .mat files (sleep) |
| 35 | ecg_sleep_std | Standard deviation of ECG amplitude (sleep) | ECG Morphology | ECG .mat files (sleep) |
| 36 | ecg_sleep_min | Minimum ECG amplitude (sleep) | ECG Morphology | ECG .mat files (sleep) |
| 37 | ecg_sleep_max | Maximum ECG amplitude (sleep) | ECG Morphology | ECG .mat files (sleep) |
| 38 | ecg_sleep_range | ECG amplitude range (sleep) | ECG Morphology | ECG .mat files (sleep) |
| 39 | ecg_sleep_snr_estimate | Signal-to-noise ratio estimate (sleep) | ECG Morphology | ECG .mat files (sleep) |
| 40 | ecg_day_length | Number of ECG samples (day recording) | ECG Morphology | ECG .mat files (day) |
| 41 | ecg_day_duration_hours | Recording duration in hours (day) | ECG Morphology | ECG .mat files (day) |
| 42 | ecg_day_mean | Mean ECG amplitude (day) | ECG Morphology | ECG .mat files (day) |
| 43 | ecg_day_std | Standard deviation of ECG amplitude (day) | ECG Morphology | ECG .mat files (day) |
| 44 | ecg_day_min | Minimum ECG amplitude (day) | ECG Morphology | ECG .mat files (day) |
| 45 | ecg_day_max | Maximum ECG amplitude (day) | ECG Morphology | ECG .mat files (day) |
| 46 | ecg_day_range | ECG amplitude range (day) | ECG Morphology | ECG .mat files (day) |
| 47 | ecg_day_snr_estimate | Signal-to-noise ratio estimate (day) | ECG Morphology | ECG .mat files (day) |
| 48 | hrv_ds_mean_rr | Mean RR interval (Deep Sleep (N3)) | HRV Time-Domain | RR-interval .mat (DS) |
| 49 | hrv_ds_std_rr | SDNN - SD of RR intervals (Deep Sleep (N3)) | HRV Time-Domain | RR-interval .mat (DS) |
| 50 | hrv_ds_mean_hr | Mean heart rate in bpm (Deep Sleep (N3)) | HRV Time-Domain | RR-interval .mat (DS) |
| 51 | hrv_ds_rmssd | RMSSD of successive differences (Deep Sleep (N3)) | HRV Time-Domain | RR-interval .mat (DS) |
| 52 | hrv_ds_pnn50 | Percentage of successive RR >50ms (Deep Sleep (N3)) | HRV Time-Domain | RR-interval .mat (DS) |
| 53 | hrv_ds_min_rr | Minimum RR interval (Deep Sleep (N3)) | HRV Time-Domain | RR-interval .mat (DS) |
| 54 | hrv_ds_max_rr | Maximum RR interval (Deep Sleep (N3)) | HRV Time-Domain | RR-interval .mat (DS) |
| 55 | hrv_ds_range_rr | RR interval range (Deep Sleep (N3)) | HRV Time-Domain | RR-interval .mat (DS) |
| 56 | hrv_ds_duration_hours | Total stage duration in hours (Deep Sleep (N3)) | HRV Time-Domain | RR-interval .mat (DS) |
| 57 | hrv_ds_count | Number of RR intervals (Deep Sleep (N3)) | HRV Time-Domain | RR-interval .mat (DS) |
| 58 | hrv_ds_cv | Coefficient of variation of RR (Deep Sleep (N3)) | HRV Time-Domain | RR-interval .mat (DS) |
| 59 | hrv_rs_mean_rr | Mean RR interval (Stable Sleep) | HRV Time-Domain | RR-interval .mat (RS) |
| 60 | hrv_rs_std_rr | SDNN - SD of RR intervals (Stable Sleep) | HRV Time-Domain | RR-interval .mat (RS) |
| 61 | hrv_rs_mean_hr | Mean heart rate in bpm (Stable Sleep) | HRV Time-Domain | RR-interval .mat (RS) |
| 62 | hrv_rs_rmssd | RMSSD of successive differences (Stable Sleep) | HRV Time-Domain | RR-interval .mat (RS) |
| 63 | hrv_rs_pnn50 | Percentage of successive RR >50ms (Stable Sleep) | HRV Time-Domain | RR-interval .mat (RS) |
| 64 | hrv_rs_min_rr | Minimum RR interval (Stable Sleep) | HRV Time-Domain | RR-interval .mat (RS) |
| 65 | hrv_rs_max_rr | Maximum RR interval (Stable Sleep) | HRV Time-Domain | RR-interval .mat (RS) |
| 66 | hrv_rs_range_rr | RR interval range (Stable Sleep) | HRV Time-Domain | RR-interval .mat (RS) |
| 67 | hrv_rs_duration_hours | Total stage duration in hours (Stable Sleep) | HRV Time-Domain | RR-interval .mat (RS) |
| 68 | hrv_rs_count | Number of RR intervals (Stable Sleep) | HRV Time-Domain | RR-interval .mat (RS) |
| 69 | hrv_rs_cv | Coefficient of variation of RR (Stable Sleep) | HRV Time-Domain | RR-interval .mat (RS) |
| 70 | hrv_rem_mean_rr | Mean RR interval (REM Sleep) | HRV Time-Domain | RR-interval .mat (REM) |
| 71 | hrv_rem_std_rr | SDNN - SD of RR intervals (REM Sleep) | HRV Time-Domain | RR-interval .mat (REM) |
| 72 | hrv_rem_mean_hr | Mean heart rate in bpm (REM Sleep) | HRV Time-Domain | RR-interval .mat (REM) |
| 73 | hrv_rem_rmssd | RMSSD of successive differences (REM Sleep) | HRV Time-Domain | RR-interval .mat (REM) |
| 74 | hrv_rem_pnn50 | Percentage of successive RR >50ms (REM Sleep) | HRV Time-Domain | RR-interval .mat (REM) |
| 75 | hrv_rem_min_rr | Minimum RR interval (REM Sleep) | HRV Time-Domain | RR-interval .mat (REM) |
| 76 | hrv_rem_max_rr | Maximum RR interval (REM Sleep) | HRV Time-Domain | RR-interval .mat (REM) |
| 77 | hrv_rem_range_rr | RR interval range (REM Sleep) | HRV Time-Domain | RR-interval .mat (REM) |
| 78 | hrv_rem_duration_hours | Total stage duration in hours (REM Sleep) | HRV Time-Domain | RR-interval .mat (REM) |
| 79 | hrv_rem_count | Number of RR intervals (REM Sleep) | HRV Time-Domain | RR-interval .mat (REM) |
| 80 | hrv_rem_cv | Coefficient of variation of RR (REM Sleep) | HRV Time-Domain | RR-interval .mat (REM) |
| 81 | hrv_ds_mean_rr_age_normalized | Age-normalized mean RR (Deep Sleep); formula: mean_RR / (age/65 + 0.1) | Age-Normalized HRV | Derived (computed) |
| 82 | hrv_rs_mean_rr_age_normalized | Age-normalized mean RR (Stable Sleep); formula: mean_RR / (age/65 + 0.1) | Age-Normalized HRV | Derived (computed) |
| 83 | hrv_rem_mean_rr_age_normalized | Age-normalized mean RR (REM); formula: mean_RR / (age/65 + 0.1) | Age-Normalized HRV | Derived (computed) |
| 84 | psqi_age | Age (from PSQI form) — duplicate of demographics | Sleep Quality (PSQI) | Objective sleep quality.xlsx |
| 85 | psqi_height | Height (from PSQI form) — duplicate of demographics | Sleep Quality (PSQI) | Objective sleep quality.xlsx |
| 86 | psqi_weight | Weight (from PSQI form) — duplicate of demographics | Sleep Quality (PSQI) | Objective sleep quality.xlsx |
| 87 | psqi_subjective sleep quality | PSQI Component 1: subjective sleep quality (0–3) | Sleep Quality (PSQI) | Objective sleep quality.xlsx |
| 88 | psqi_sleep latency | PSQI Component 2: sleep latency (0–3) | Sleep Quality (PSQI) | Objective sleep quality.xlsx |
| 89 | psqi_sleep duration | PSQI Component 3: sleep duration (0–3) | Sleep Quality (PSQI) | Objective sleep quality.xlsx |
| 90 | psqi_habitual sleep efficiency | PSQI Component 4: habitual sleep efficiency (0–3) | Sleep Quality (PSQI) | Objective sleep quality.xlsx |
| 91 | psqi_sleep disturbances | PSQI Component 5: sleep disturbances (0–3) | Sleep Quality (PSQI) | Objective sleep quality.xlsx |
| 92 | psqi_sleep medication | PSQI Component 6: use of sleep medication (0–3) | Sleep Quality (PSQI) | Objective sleep quality.xlsx |
| 93 | psqi_daytime dysfunction | PSQI Component 7: daytime dysfunction (0–3) | Sleep Quality (PSQI) | Objective sleep quality.xlsx |
| 94 | psqi_PSQI score | Global PSQI score (0–21; >5 = poor sleep quality) | Sleep Quality (PSQI) | Objective sleep quality.xlsx |
| 95 | cpc_AHI (event per hour) | Apnea-hypopnea index | Sleep Quality (CPC) | Subjective sleep quality.xlsx |
| 96 | cpc_TST (hour) | Total sleep time | Sleep Quality (CPC) | Subjective sleep quality.xlsx |
| 97 | cpc_UST (hour) | Unstable sleep time | Sleep Quality (CPC) | Subjective sleep quality.xlsx |
| 98 | cpc_SST (hour) | Stable sleep time | Sleep Quality (CPC) | Subjective sleep quality.xlsx |
| 99 | cpc_RST (hour) | REM sleep time | Sleep Quality (CPC) | Subjective sleep quality.xlsx |
| 100 | cpc_ALUS (min) | Average length of unstable sleep segments | Sleep Quality (CPC) | Subjective sleep quality.xlsx |
| 101 | cpc_ALSS (min) | Average length of stable sleep segments | Sleep Quality (CPC) | Subjective sleep quality.xlsx |
| 102 | cpc_ALRS (min) | Average length of REM sleep segments | Sleep Quality (CPC) | Subjective sleep quality.xlsx |
| 103 | cpc_USP (%) | Unstable sleep percentage of TST | Sleep Quality (CPC) | Subjective sleep quality.xlsx |
| 104 | cpc_SSP (%) | Stable sleep percentage of TST | Sleep Quality (CPC) | Subjective sleep quality.xlsx |
| 105 | cpc_RSP (%) | REM sleep percentage of TST | Sleep Quality (CPC) | Subjective sleep quality.xlsx |

***Note:*** *Features are listed in extraction order. Domain colour coding corresponds to Figure 5 of manuscript (feature importance by domain). DS = Deep/Unstable Sleep (N3); RS = Stable/Rapid Sleep; REM = Rapid Eye Movement sleep. Sleep stage terminology follows the CPC analysis convention used in the source dataset. PSQI = Pittsburgh Sleep Quality Index; CPC = Cardiopulmonary Coupling analysis. The 11 excluded columns (subject_id, gender, admission FBG, discharge FBG, HbA1c, and 6 diabetic complication indicators) are not shown.*

### 2. Sensitivity Analysis (Age Normalization)

To evaluate the robustness of our proposed age-normalization formula, we performed a grid search analysis on the two hyperparameters: $Age Threshold (T)$and $Smoothing Factor(\varepsilon)$.The normalization formula is defined as:

${HRV}_{normalized}$ = $\frac{\mathrm{HRV}_{\mathrm{raw}}}{(\frac{\mathrm{age}}{T} + \varepsilon)}$

We tested 20 combinations of parameters $\left( T \in\left\{ 55, 60, 65, 70, 75 \right\} \right), \varepsilon\in\left\{ 0.05, 0.10, 0.15, 0.20 \right\}$ to observe their impact on the model's predictive performance $\left( R^{2} \right)$.

**2.1 HbA1c Cohort Results**

The sensitivity analysis as shown in Table S2 and Figure 2, demonstrates that the physiological signal is highly robust to parameter selection.

1. Optimal Region: While the final optimized Extra Trees model achieved an $\left( R^{2}=0.222 \right)$ (Table S7), the grid search on the baseline estimator confirms that performance remains stable across the majority of parameter combinations $\left( T \in\left\{ 55, 60, 65, 70, 75 \right\} \right)$.
2. Stability: Deviations in $\varepsilon$. resulted in negligible changes to the Mean Absolute Error (MAE), typically fluctuating by less than 0.005 units. This indicates that the normalization successfully captures the physiological interaction between age and HRV without introducing volatility.


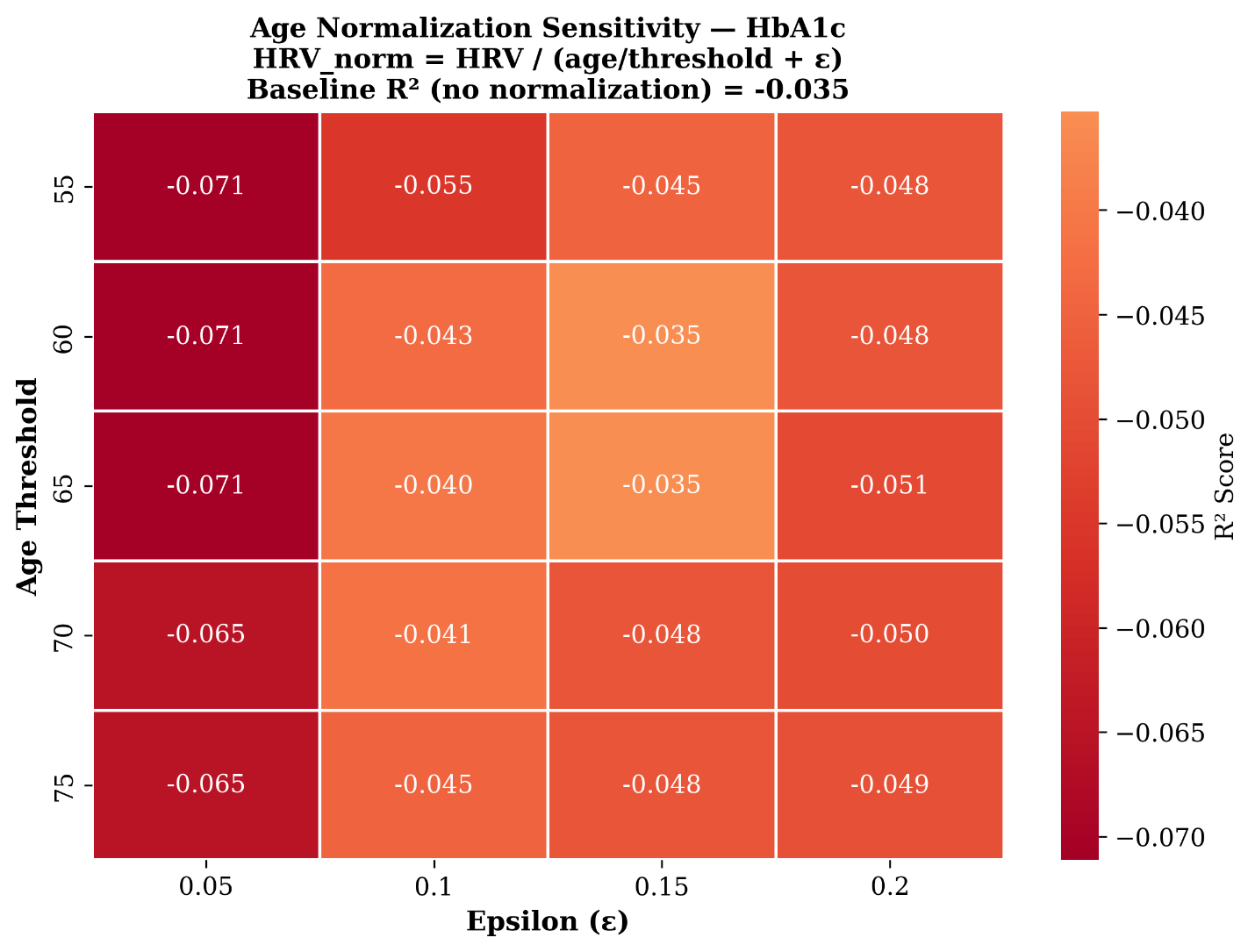


**Figure 2** Age Sensitivity Heatmap (HbA1c)

**Table S2** Sensitivity analysis of age normalization parameters (HbA1c Cohort)

| *Threshold (T)* | $\boldsymbol{\epsilon}$ | $\boldsymbol{R}^{\boldsymbol{2}}$ | $\boldsymbol{MAE}$ | $\boldsymbol{\Delta R}^{\boldsymbol{2}}$ | *Normalization Method* |
| --- | --- | --- | --- | --- | --- |
| None | None | -0.035 | 0.169 | 0.000 | No normalization (baseline) |
| 55 | 0.05 | -0.071 | 0.173 | -0.036 | $\frac{\mathrm{HRV}_{\mathrm{raw}}}{(\frac{\mathrm{age}}{55} + 0.05)}$ |
| 55 | 0.1 | -0.055 | 0.172 | -0.020 | $\frac{\mathrm{HRV}_{\mathrm{raw}}}{(\frac{\mathrm{age}}{55} + 0.1)}$ |
| 55 | 0.15 | -0.045 | 0.171 | -0.009 | $\frac{\mathrm{HRV}_{\mathrm{raw}}}{(\frac{\mathrm{age}}{55} + 0.15)}$ |
| 55 | 0.2 | -0.048 | 0.172 | -0.013 | $\frac{\mathrm{HRV}_{\mathrm{raw}}}{(\frac{\mathrm{age}}{55} + 0.2)}$ |
| 60 | 0.05 | -0.071 | 0.173 | -0.036 | $\frac{\mathrm{HRV}_{\mathrm{raw}}}{(\frac{\mathrm{age}}{60} + 0.05)}$ |
| 60 | 0.1 | -0.043 | 0.170 | -0.008 | $\frac{\mathrm{HRV}_{\mathrm{raw}}}{(\frac{\mathrm{age}}{60} + 0.1)}$ |
| 60 | 0.15 | -0.035 | 0.170 | 0.000 | $\frac{\mathrm{HRV}_{\mathrm{raw}}}{(\frac{\mathrm{age}}{60} + 0.15)}$ |
| 60 | 0.2 | -0.048 | 0.172 | -0.013 | $\frac{\mathrm{HRV}_{\mathrm{raw}}}{(\frac{\mathrm{age}}{60} + 0.2)}$ |
| 65 | 0.05 | -0.071 | 0.173 | -0.036 | $\frac{\mathrm{HRV}_{\mathrm{raw}}}{(\frac{\mathrm{age}}{65} + 0.05)}$ |
| 65 | 0.1 | -0.040 | 0.170 | -0.005 | $\frac{\mathrm{HRV}_{\mathrm{raw}}}{(\frac{\mathrm{age}}{65} + 0.1)}$ |
| 65 | 0.15 | -0.035 | 0.170 | 0.000 | $\frac{\mathrm{HRV}_{\mathrm{raw}}}{(\frac{\mathrm{age}}{65} + 0.15)}$ |
| 65 | 0.2 | -0.051 | 0.172 | -0.015 | $\frac{\mathrm{HRV}_{\mathrm{raw}}}{(\frac{\mathrm{age}}{65} + 0.2)}$ |
| 70 | 0.05 | -0.065 | 0.172 | -0.030 | $\frac{\mathrm{HRV}_{\mathrm{raw}}}{(\frac{\mathrm{age}}{70} + 0.05)}$ |
| 70 | 0.1 | -0.041 | 0.170 | -0.006 | $\frac{\mathrm{HRV}_{\mathrm{raw}}}{(\frac{\mathrm{age}}{70} + 0.1)}$ |
| 70 | 0.15 | -0.048 | 0.171 | -0.012 | $\frac{\mathrm{HRV}_{\mathrm{raw}}}{(\frac{\mathrm{age}}{70} + 0.15)}$ |
| 70 | 0.2 | -0.050 | 0.172 | -0.015 | $\frac{\mathrm{HRV}_{\mathrm{raw}}}{(\frac{\mathrm{age}}{70} + 0.2)}$ |
| 75 | 0.05 | -0.065 | 0.172 | -0.030 | $\frac{\mathrm{HRV}_{\mathrm{raw}}}{(\frac{\mathrm{age}}{75} + 0.05)}$ |
| 75 | 0.1 | -0.045 | 0.171 | -0.009 | $\frac{\mathrm{HRV}_{\mathrm{raw}}}{(\frac{\mathrm{age}}{75} + 0.1)}$ |
| 75 | 0.15 | -0.048 | 0.172 | -0.013 | $\frac{\mathrm{HRV}_{\mathrm{raw}}}{(\frac{\mathrm{age}}{75} + 0.15)}$ |
| 75 | 0.2 | -0.049 | 0.172 | -0.014 | $\frac{\mathrm{HRV}_{\mathrm{raw}}}{(\frac{\mathrm{age}}{75} + 0.2)}$ |

**2.2 FBG Cohort Results**

In contrast, the FBG cohort showed higher sensitivity and a different response to normalization as shown in Table S3 and Figure 3.

1. Performance: The baseline (non-normalized) model performed comparably to, or slightly better than, the age-normalized versions (Table S7).
2. Interpretation: This suggests that for instantaneous Fasting Blood Glucose, the direct physiological link between Age and HRV is less dominant than it is for long-term HbA1c, or that the short-term variability of FBG masks the age-dependent signal.


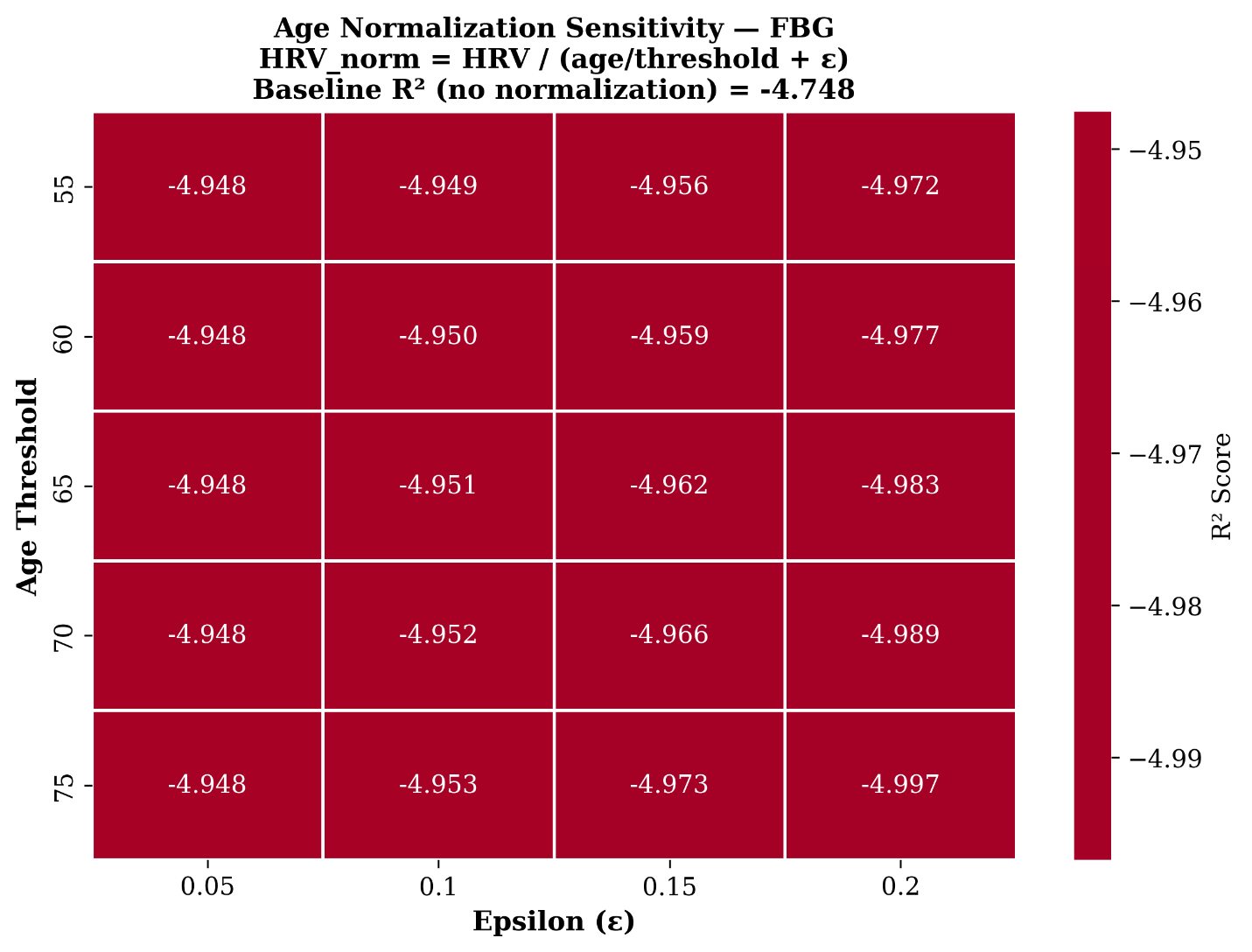


**Figure 3** Age Sensitivity Heatmap (FBG)

**Table S3** Sensitivity analysis of age normalization parameters (FBG Cohort)

| *Threshold (T)* | $\boldsymbol{\epsilon}$ | $\boldsymbol{R}^{\boldsymbol{2}}$ | $\boldsymbol{MAE}$ | $\boldsymbol{\Delta}\boldsymbol{R}^{\boldsymbol{2}}$ | *Normalization Method* |
| --- | --- | --- | --- | --- | --- |
| None | None | -4.75 | 0.360 | 0.000 | No normalization (baseline) |
| 55 | 0.05 | -4.95 | 0.364 | -0.201 | $\frac{\mathrm{HRV}_{\mathrm{raw}}}{(\frac{\mathrm{age}}{55} + 0.05)}$ |
| 55 | 0.1 | -4.95 | 0.365 | -0.201 | $\frac{\mathrm{HRV}_{\mathrm{raw}}}{(\frac{\mathrm{age}}{55} + 0.1)}$ |
| 55 | 0.15 | -4.96 | 0.365 | -0.208 | $\frac{\mathrm{HRV}_{\mathrm{raw}}}{(\frac{\mathrm{age}}{55} + 0.15)}$ |
| 55 | 0.2 | -4.97 | 0.366 | -0.224 | $\frac{\mathrm{HRV}_{\mathrm{raw}}}{(\frac{\mathrm{age}}{55} + 0.2)}$ |
| 60 | 0.05 | -4.95 | 0.364 | -0.200 | $\frac{\mathrm{HRV}_{\mathrm{raw}}}{(\frac{\mathrm{age}}{60} + 0.05)}$ |
| 60 | 0.1 | -4.95 | 0.365 | -0.202 | $\frac{\mathrm{HRV}_{\mathrm{raw}}}{(\frac{\mathrm{age}}{60} + 0.1)}$ |
| 60 | 0.15 | -4.96 | 0.365 | -0.211 | $\frac{\mathrm{HRV}_{\mathrm{raw}}}{(\frac{\mathrm{age}}{60} + 0.15)}$ |
| 60 | 0.2 | -4.98 | 0.366 | -0.229 | $\frac{\mathrm{HRV}_{\mathrm{raw}}}{(\frac{\mathrm{age}}{60} + 0.2)}$ |
| 65 | 0.05 | -4.95 | 0.364 | -0.200 | $\frac{\mathrm{HRV}_{\mathrm{raw}}}{(\frac{\mathrm{age}}{65} + 0.05)}$ |
| 65 | 0.1 | -4.95 | 0.365 | -0.203 | $\frac{\mathrm{HRV}_{\mathrm{raw}}}{(\frac{\mathrm{age}}{65} + 0.1)}$ |
| 65 | 0.15 | -4.96 | 0.365 | -0.214 | $\frac{\mathrm{HRV}_{\mathrm{raw}}}{(\frac{\mathrm{age}}{65} + 0.15)}$ |
| 65 | 0.2 | -4.98 | 0.366 | -0.236 | $\frac{\mathrm{HRV}_{\mathrm{raw}}}{(\frac{\mathrm{age}}{65} + 0.2)}$ |
| 70 | 0.05 | -4.95 | 0.364 | -0.200 | $\frac{\mathrm{HRV}_{\mathrm{raw}}}{(\frac{\mathrm{age}}{70} + 0.05)}$ |
| 70 | 0.1 | -4.95 | 0.365 | -0.204 | $\frac{\mathrm{HRV}_{\mathrm{raw}}}{(\frac{\mathrm{age}}{70} + 0.1)}$ |
| 70 | 0.15 | -4.97 | 0.365 | -0.218 | $\frac{\mathrm{HRV}_{\mathrm{raw}}}{(\frac{\mathrm{age}}{70} + 0.15)}$ |
| 70 | 0.2 | -4.99 | 0.366 | -0.242 | $\frac{\mathrm{HRV}_{\mathrm{raw}}}{(\frac{\mathrm{age}}{70} + 0.2)}$ |
| 75 | 0.05 | -4.95 | 0.364 | -0.200 | $\frac{\mathrm{HRV}_{\mathrm{raw}}}{(\frac{\mathrm{age}}{75} + 0.05)}$ |
| 75 | 0.1 | -4.95 | 0.365 | -0.206 | $\frac{\mathrm{HRV}_{\mathrm{raw}}}{(\frac{\mathrm{age}}{75} + 0.1)}$ |
| 75 | 0.15 | -4.97 | 0.366 | -0.225 | $\frac{\mathrm{HRV}_{\mathrm{raw}}}{(\frac{\mathrm{age}}{75} + 0.15)}$ |
| 75 | 0.2 | -5.00 | 0.367 | -0.249 | $\frac{\mathrm{HRV}_{\mathrm{raw}}}{(\frac{\mathrm{age}}{75} + 0.2)}$ |

**3. Clinical Interpretation of Error Metrics**

The Table S4 displays the predictive performance of the final Extra Trees regression model on the original clinical scale. MAE represents the average magnitude of the error in clinical units (% for HbA1c, mmol/L for FBG).

**Table S4** Back-transformed prediction performance on clinical scales

| *Cohort* | *Clinical Unit* | *MAE* | *RMSE* | *Median AE* | *MAPE (%)* |
| --- | --- | --- | --- | --- | --- |
| HbA1c | % | 1.18 | 1.44 | 1.15 | 14.6% |
| FBG | mmol/L | 2.27 | 2.98 | 1.46 | 24.9% |

**4. Feature Selection Stability Analysis**

To ensure that the identified biomarkers are not artifacts of a specific data split, we analyzed the stability of feature selection across the Leave-One-Subject-Out (LOSO) cross-validation folds. A feature is considered "Stable" if it is selected by the model in >80% of the training folds.

**4.1 HbA1c Cohort**

Feature stability in the HbA1c cohort (N=29) highlights metabolic traits as shown in Table S5.

- **100% Stability:** Age, DBP (Diastolic Blood Pressure), Hb (Hemoglobin), and liver enzymes (AST/ALT, ALT) were selected in every single fold.
- **HRV Signal:** The age-normalized HRV feature (hrv_ds_mean_rr_age_normalized) achieved **93% stability**, confirming it is a consistent predictor of HbA1c alongside traditional metabolic markers.

**Table S5** Feature selection stability for the HbA1c cohort (N=29)

| *Feature* | *Folds_Selected* | *Selection_Rate* | *Stability* |
| --- | --- | --- | --- |
| age | 29 | 1.00 | Stable |
| DBP (mmHg) | 29 | 1.00 | Stable |
| Hb (g/L) | 29 | 1.00 | Stable |
| ALT (U/L) | 29 | 1.00 | Stable |
| AST/ALT | 29 | 1.00 | Stable |
| psqi_age | 29 | 1.00 | Stable |
| GGT (U/L) | 27 | 0.93 | Stable |
| psqi_habitual sleep efficiency | 27 | 0.93 | Stable |
| hrv_ds_mean_rr_age_normalized | 27 | 0.93 | Stable |
| psqi_sleep disturbances | 26 | 0.90 | Stable |
| hrv_rem_mean_rr_age_normalized | 26 | 0.90 | Stable |
| weight | 23 | 0.79 | Moderate |
| psqi_subjective sleep quality | 23 | 0.79 | Moderate |
| hrv_rs_mean_rr_age_normalized | 23 | 0.79 | Moderate |
| TG (mmol/L) | 22 | 0.76 | Moderate |
| ecg_day_snr_estimate | 10 | 0.34 | Unstable |
| hrv_ds_rmssd | 6 | 0.21 | Unstable |
| ecg_day_min | 5 | 0.17 | Unstable |
| ecg_sleep_length | 2 | 0.07 | Unstable |
| ecg_sleep_duration_hours | 2 | 0.07 | Unstable |
| psqi_PSQI score | 2 | 0.07 | Unstable |
| UACR (mg/g) | 1 | 0.034 | Unstable |
| BUN (mmol/L) | 1 | 0.034 | Unstable |
| LDL-C (mmol/L) | 1 | 0.034 | Unstable |
| hrv_ds_mean_hr | 1 | 0.034 | Unstable |
| hrv_ds_cv | 1 | 0.034 | Unstable |
| hrv_rs_mean_hr | 1 | 0.034 | Unstable |
| hrv_rem_mean_hr | 1 | 0.034 | Unstable |
| psqi_weight | 1 | 0.034 | Unstable |
| WBC $\left( \boldsymbol{X}\boldsymbol{10}^{\boldsymbol{9}}\boldsymbol{/L} \right)$ | 1 | 0.034 | Unstable |
| height | 1 | 0.034 | Unstable |

**4.2 FBG Cohort**

Feature stability in the FBG cohort (N=38) reveals a shift toward state-dependent variables as shown in Table S6.

- **Sleep Duration:** Unlike the HbA1c cohort, ecg_sleep_duration emerged as a 100% stable predictor for FBG.
- **Interpretation:** This distinction suggests that while HbA1c is driven by stable physiological traits (liver function, age), instantaneous fasting glucose is significantly influenced by the immediate "state" of the subject, specifically the duration of rest prior to measurement.

**Table S6** Feature selection stability for the FBG cohort (N=38)

| *Feature* | *Folds_Selected* | *Selection_Rate* | *Stability* |
| --- | --- | --- | --- |
| age | 38 | 1 | Stable |
| DBP (mmHg) | 38 | 1 | Stable |
| Hb (g/L) | 38 | 1 | Stable |
| ecg_sleep_length | 38 | 1 | Stable |
| ecg_sleep_duration_hours | 38 | 1 | Stable |
| psqi_age | 38 | 1 | Stable |
| hrv_ds_mean_rr_age_normalized | 38 | 1 | Stable |
| hrv_rem_mean_rr_age_normalized | 38 | 1 | Stable |
| hrv_rs_mean_rr_age_normalized | 37 | 0.974 | Stable |
| ecg_all_min | 36 | 0.947 | Stable |
| ecg_all_range | 33 | 0.868 | Stable |
| LDL-C (mmol/L) | 30 | 0.789 | Moderate |
| psqi_weight | 29 | 0.763 | Moderate |
| TG (mmol/L) | 28 | 0.737 | Moderate |
| weight | 20 | 0.526 | Moderate |
| ecg_sleep_range | 16 | 0.421 | Unstable |
| hrv_rem_rmssd | 8 | 0.211 | Unstable |
| ecg_sleep_max | 4 | 0.105 | Unstable |
| ALT (U/L) | 4 | 0.105 | Unstable |
| ecg_sleep_min | 2 | 0.053 | Unstable |
| psqi_sleep disturbances | 2 | 0.053 | Unstable |
| hrv_ds_count | 2 | 0.053 | Unstable |
| hrv_ds_rmssd | 2 | 0.053 | Unstable |
| hrv_rem_pnn50 | 2 | 0.053 | Unstable |
| psqi_sleep medication | 1 | 0.026 | Unstable |
| ecg_day_min | 1 | 0.026 | Unstable |
| AST/ALT | 1 | 0.026 | Unstable |
| hrv_ds_duration_hours | 1 | 0.026 | Unstable |
| BUN (mmol/L) | 1 | 0.026 | Unstable |
| hrv_rem_max_rr | 1 | 0.026 | Unstable |
| hrv_rem_range_rr | 1 | 0.026 | Unstable |
| PLT$\left( \boldsymbol{X}\boldsymbol{10}^{\boldsymbol{9}}\boldsymbol{/L} \right)$ | 1 | 0.026 | Unstable |
| ecg_all_mean | 1 | 0.026 | Unstable |
| ecg_all_max | 1 | 0.026 | Unstable |
| hrv_ds_pnn50 | 1 | 0.026 | Unstable |

**5. Baseline Model Comparison**

To benchmark the performance of the proposed method, we compared the Extra Trees Regressor against a comprehensive suite of 20 standard machine learning algorithms. The analysis shown in Table S7, included linear baselines, support vector machines (SVM), and ensemble methods.

**Table S7** Full baseline model comparison (20 Models).

| *Model* | *HbA1c* | *HbA1c* | *HbA1c* | *FBG* | *FBG* | *FBG* |
| --- | --- | --- | --- | --- | --- | --- |
|  | $\boldsymbol{R}^{\boldsymbol{2}}$ | ***MAE*** | ***RMSE*** | $\boldsymbol{R}^{\boldsymbol{2}}$ | ***MAE*** | ***RMSE*** |
| Naive (Mean) | -0.073 | 0.169 | 0.205 | -0.055 | 0.287 | 0.332 |
| Naive (Median) | -0.027 | 0.165 | 0.201 | -0.066 | 0.290 | 0.334 |
| Linear Regression | -13.698 | 0.365 | 0.760 | -59.141 | 0.741 | 2.510 |
| Ridge $\left( \boldsymbol{\alpha=1.0} \right)$ | -0.281 | 0.195 | 0.224 | -0.073 | 0.264 | 0.335 |
| Ridge $\left( \boldsymbol{\alpha=0.1} \right)$ | -0.804 | 0.230 | 0.266 | -9.106 | 0.421 | 1.029 |
| Lasso $\left( \boldsymbol{\alpha=0.1} \right)$ | -0.051 | 0.169 | 0.203 | 0.005 | 0.269 | 0.323 |
| ElasticNet | 0.049 | 0.163 | 0.193 | 0.031 | 0.261 | 0.319 |
| Bayesian Ridge | -0.035 | 0.169 | 0.202 | -4.748 | 0.360 | 0.776 |
| Huber Regressor | -11.659 | 0.371 | 0.705 | -2.932 | 0.391 | 0.642 |
| Random Forest | 0.027 | 0.164 | 0.196 | 0.047 | 0.255 | 0.316 |
| Extra Trees | 0.222 | 0.142 | 0.175 | 0.086 | 0.247 | 0.309 |
| Gradient Boosting | -0.114 | 0.153 | 0.209 | -0.048 | 0.263 | 0.331 |
| AdaBoost | -0.253 | 0.178 | 0.222 | -0.330 | 0.304 | 0.373 |
| SVR (RBF) | 0.016 | 0.168 | 0.197 | -0.174 | 0.288 | 0.351 |
| SVR (Linear) | -0.299 | 0.193 | 0.226 | -17.462 | 0.493 | 1.391 |
| SVR (Poly) | -0.651 | 0.213 | 0.255 | -123507.552 | 18.872 | 113.733 |
| MLP (32) | -8.162 | 0.485 | 0.600 | -10878.857 | 5.975 | 33.756 |
| MLP (64, 32) | -18.178 | 0.684 | 0.868 | -626.696 | 2.150 | 8.108 |
| MLP (128, 64, 32) | -23.413 | 0.735 | 0.980 | -5450.049 | 4.638 | 23.893 |
| MLP (64, 32) tanh | -24.636 | 0.649 | 1.004 | -10.140 | 0.728 | 1.080 |
